# Inter-Gender sEMG Evaluation of Central and Peripheral Fatigue in Biceps Brachii of Young Healthy Subjects

**DOI:** 10.1371/journal.pone.0168443

**Published:** 2016-12-21

**Authors:** Federico Meduri, Matteo Beretta-Piccoli, Luca Calanni, Valentina Segreto, Giuseppe Giovanetti, Marco Barbero, Corrado Cescon, Giuseppe D’Antona

**Affiliations:** 1 Department of Public Health, Molecular and Forensic Medicine, and Sport Medicine Centre Voghera, University of Pavia, Pavia, Italy; 2 Rehabilitation Research Laboratory 2rLab, Department of Business Economics, Health and Social Care, University of Applied Sciences and Arts of Southern Switzerland, Manno, Switzerland; University of Houston, UNITED STATES

## Abstract

**Purpose:**

The purpose of the present study was to evaluate inter-arm and inter-gender differences in fractal dimension (FD) and conduction velocity (CV) obtained from multichannel surface electromyographic (sEMG) recordings during sustained fatiguing contractions of the biceps brachii.

**Methods:**

A total of 20 recreationally active males (24±6 years) and 18 recreationally active females (22±9 years) performed two isometric contractions at 120 degrees elbow joint angle: (1) at 20% maximal voluntary contraction (MVC) for 90 s, and (2) at 60% MVC until exhaustion the time to perform the task has been measured. Signals from sEMG were detected from the biceps brachii using bidimensional arrays of 64 electrodes and initial values and rate of change of CV and FD of the sEMG signal were calculated.

**Results:**

No difference between left and right sides and no statistically significant interaction effect of sides with gender were found for all parameters measured. A significant inter-gender difference was found for MVC (p<0.0001). Initial values of CV were higher in females than in males at both force levels (20% MCV: p<0.0001; 60% MCV: p<0.05) whereas a lower initial estimate of FD was observed in females compared to males (20% MCV: p<0.05; 60% MCV: p<0.0001). No difference in CV and FD slopes was found at 20% MVC between genders. At 60% MVC significantly lower CV and FD slopes (CV and FD: p<0.05) and a more protracted time to exhaustion were found in females than in males (p<0.0001). When considering time to exhaustion at both levels of contraction no difference in percentage change (**Δ%**) of CV and FD slopes was found between genders (p>0.05). During the sustained 60% MVC no statistical correlation was found between MVC and CV or FD initial estimates nor between MVC and CV or FD slopes both in males and females whereas. A significant positive correlation between CV and FD slopes was found in both genders (males: r = 0,61; females: r = 0,55).

**Conclusions:**

Fatigue determines changes in FD and CV values in biceps brachii during sustained contractions at 60% MVC. In particular males show greater increase in the rate of change of CV and FD than females whereas no difference in percentage change of these sEMG descriptors of fatigue was found. A significant correlation between FD and CV slopes found in both genders highlights that central and peripheral myoelectric components of fatigue may interact during submaximal isometric contractions.

## Introduction

Physiological fatigue is an experience of daily life responsible for failure to maintain the required level of force overtime [1]. Central and peripheral components lead to fatigue [2–4] but their relative contribution is not easily suitable for quantification or measurement [5].

Central fatigue originates in structures above the neuromuscular junctions from the central nervous system to the peripheral nerves leading to changes in motor unit (MU) recruitment behavior including decline in the MU recruitment threshold and progressive recruitment of new MU without change to the recruitment order [6]. Peripheral aspects of fatigue include local changes at the skeletal muscle level, namely metabolic acidosis and protons accumulation [7], that may impair sarcolemmal excitability, electro-mechanical coupling, acto-myosin myofibrillar interaction and metabolic fuelling of the myofibers.

Continuous monitoring of local muscle fatigue during a task is possible by measuring the myoelectric activity of a muscle by surface electromyography (sEMG). Biochemical and physiological changes in muscles during fatiguing contractions are, namely, reflected also in properties of myoelectric signals recorded on the surface of the skin above the muscle(s) concerned, leading to what is commonly defined as *myoelectric manifestations of muscle fatigue* [6].

Myoelectric manifestations of fatigue are mainly related to two physiological phenomena: (1) the slowing of motor unit action potentials (MUAP) during their travelling along muscle fibers, that is the reduction of their conduction velocity (CV) [8,9,10], and (2) the synchronization of MU by the central nervous system, which is defined as a higher occurrence of simultaneous discharge of action potentials from different MU to increase the mechanical output when the whole MU pool is recruited [11], as observed in trained individuals [12] and in presence of central lesions [13]. Therefore, to evaluate peripheral components of muscle fatigue, CV rate of change (i.e. slope) might be estimated during isometric tasks [14–21], whereas to evaluate central components of fatigue sEMG descriptors of MU synchronization may be useful.

The main problem with the existing techniques quantifying MU synchronization using sEMG lies in their dependency on the CV of MU action potentials (MUAPs), since MU synchronization level and CV often change in parallel [6]. Therefore, the use of nonlinear more sensitive parameters to extract information from sEMG signals during isometric and dynamic fatiguing contractions may be preferred over traditional amplitude or spectral parameters (for an exhaustive and comprehensive review, see Gonzalez-Izal et al., 2012) [16]. A sEMG descriptor of the level MU synchronization is fractal dimension (FD), which shows high dependency on MU synchronization and, compared FD to other linear and non-linear muscle fatigue indices, least affected by CV changes and weakly affected by fat layer thickness [22,23].

We recently reported that a decay of FD during sustained fatiguing isometric contractions of the quadriceps muscles may mirror a progressive MU synchronization due to central fatigue [24] in young females [25]. Indeed, Boccia and colleagues (2016) reported a steeper rate of change of FD in elderly males compared to young males during fatiguing contractions of the knee extensors suggesting a greater increase in motor unit synchronization with age [26].

In addition to CV and FD rate of change, a different method to assess central and peripheral fatigue is based on electrical stimulation during MVC combined with sEMG [27,28,29,30].

The sources of inter-individual variability in peripheral and central fatigue, namely inter-gender differences, are still elusive. Strength-matched females and males show the same time to failure of a sustained submaximal isometric contraction of the elbow flexor muscles whereas a significant sex related difference is maintained when an intermittent isometric task is performed until failure [31]. These evidence indicates that a strength-related mechanism may contribute to the longer time to task failure during isometric sustained contractions [32] and the shorter time to task exhibited by males during submaximal isometric contractions of the elbow flexors, unlike lower limb muscles [33,34], has been mainly attributed to peripheral mechanisms. These mechanisms appear to be mirrored by diverse changes in myoelectric manifestations of fatigue including higher CV slopes than females [35].

Despite females sustain isometric contractions with the elbow flexor muscles for a longer duration than males, robust evidences suggest a similar reduction in voluntary activation at task failure for superimposed stimulations at the motor cortex [36] and skeletal muscle [37]. No data are currently available on whether differences in the initial value and rate of change of MU synchronization during sustained contractions of the elbow flexors may contribute to different adaptation to muscle fatigue by the central nervous system in males compared to females. The aim of the study was to use sEMG indexes of fatigue (CV and FD) to compare healthy young males *versus* females during prolonged isometric contractions of the biceps brachii. In particular we aimed to test whether a diverse level of initial value and rate of change of MU synchronization is associated with diverse gender-related resistance to fatigue. The main conclusions of the study were: in sustained fatiguing task (1) significantly greater decrease in the rate of change of myoelectric manifestations of fatigue and higher time to task in females than males; (2) no sex difference in percentage change of CV and FD; (3) a significant correlation between CV and FD slopes at higher contraction level.

## Methods

### Subjects

A total of 20 recreationally healthy active males (mean±S.D.: age 24.95±6.1 years; height 176±4 cm; weight 73±8 kg) and 18 recreationally healthy active females all within one week after the menstrual cycle (mean±S.D: age 22±9.2 years; height 167 ±3 cm; weight 62±9 kg) reporting no clinical history of previous arms injuries, volunteered to participate. Prior to testing, subjects signed a written informed consent. The study was settled at C.R.I.A.M.S. laboratory in Voghera. The study was approved by the local ethics committee of the Swiss Italian Health and Sociality Department, Switzerland. Volunteer subjects were excluded if they had a positive history of orthopedic disorders affecting neck and shoulder regions and a positive history of neurological disorders.

### Experimental procedure

**Fig 1:**
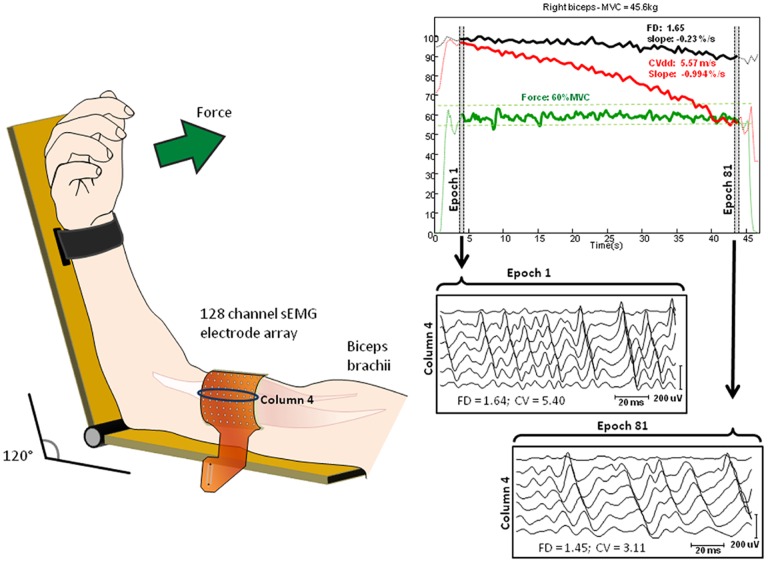
64 channels electrode arrays position on biceps brachii muscle with elbow maintained at 120 degrees angle. The electrode array is aligned with respect to the muscle fibers and located between the distal tendon and the innervation zone. On the right side example of recorded signals detected from the biceps brachii muscle of a representative subject during a 60% MVC contraction. The insets show raw surface EMG signals detected during selected epochs at the beginning and at the end of contraction.

Subjects’ arms were positioned correctly and comfortably in a isometric-ergometer (MUC1, OT Bioelettronica, Turin, Italy) equipped with a load cell (CCT Transducer, linear, full scale 100kg). In order to isolate the action of the biceps brachii, the wrist was fastened to the machine. Participants were sat, with the elbow at 120 degrees as described in Fig 1. A 64-channels matrix was placed in parallel with the orientation of the muscle fibers between the distal tendon and the innervation zone of the biceps brachii in order to have a pure propagation of MU action potentials (MUAPs). Subject’s position did not provide any support or resistance during contraction.

After 5 min rest, two isometric MVCs were performed, separated by 2 min rest. During each contraction, the force trace was displayed on a computer monitor as visual feedback. Participants were instructed to increase the force up to the maximum, and to hold it as steady as possible, for 2–3 s. Participants were given verbal encouragement.

After 2 min rest, a low intensity sustained contraction (20% MVC) was performed for 90s and the Borg scale values (from 6 to 20) [38] were obtained. After 4 min rest, subjects were asked to perform a high level sustained contraction (60% MVC) until exhaustion, during which they were verbally encouraged to keep the force level as long as possible, until the force value decreased to below 90% of the target [39]. The experimental procedure was randomly conducted on both arms (30 min rest between side) in two trials, with an interval of a week.

### EMG and force measurements

Myoelectric signals were detected through a 64 electrodes matrix (10 mm IED, 8 lines, 8 columns) (Spes Medica, Battipaglia, Italy) used to obtain monopolar sEMG signals. Biceps brachii was chosen primarily to obtain high-quality sEMG signals due to the simplicity of movement, isolation of the muscle contraction, and parallel fiber orientation. The adhesive matrix was applied following muscle fibers leanings in correspondence to the muscle belly previously identified by ultrasound scan (Phillips CX-30). The advantage of multichannel acquisition is the possibility to select the best channels afterwards, reducing the risk of short circuits or bad contacts. The EMG signals were amplified (EMG-USB2; OT Bioelettronica, Turin, Italy), sampled at 2048 Hz, and stored on a computer.

### Signal processing

Data were divided in epochs of 0.5 seconds and each variable was computed for each epoch. The exhaustion time was computed as the time instant when the force was below 5% of the target. The regression line was computed for all the values from the beginning of the contraction to the exhaustion time. For each acquisition, we selected the channels for the analysis by means of visual inspection. The column showing the largest portion of propagating channels with biggest amplitude was selected, and the channels between IZ and tendons were selected for CV computation. FD was computed for each of the selected channels and then averaged. FD was estimated using the box-counting method. Briefly, as expressed in [40], a grid of square boxes was used to cover the signal, and the number of boxes that the sEMG waveform passes through was counted. When decreasing the side of the boxes in a dichotomic process, the number of boxes required to cover the signal increased exponentially. However, by plotting the logarithm of the number of boxes counted (log N) vs. the logarithm of the inverse of the box size (log 1/S), the exponential relationship became linear. The slope of the interpolation line (estimated using the least mean squared procedure) is the FD. CV was estimated using a multichannel algorithm on double differential signals, based on the matching between signals filtered in the temporal and in the spatial domains [41].

CV values outside the physiological range (3–6 m/s) [42], were excluded from the analysis.

### Statistical analyses

The variables used for the statistics were the initial values and slopes of CV and FD, determined by linear regression overtime.

A Shapiro-Wilk test revealed that the variables were normally distributed. Data were reported as mean ± standard deviation (SD) and statistical significance was set to P<0.05. Paired t-test was used to compare significant differences between arms, unpaired t-test was used to compare significant differences between genders, whereas two-way ANOVA for multiple comparisons was used to find significant interactions between side and gender. Statistical analyses were performed using Prism Graphpad (San Diego, California). Statistical tests were conducted for FD and muscle fiber CV initial values and slopes, considering data from left and right biceps brachii as a single set.

## Results

For all parameters measured no statistical difference was found between right and left arm, and no significant two-way interaction effect was found between arm side (left and right) and gender (male and female). Therefore data from left and right biceps brachii were pooled for subsequent analysis. In females, biceps brachii was characterized by thicker skinfold than in males (females 7.3 ± 2.6 mm; males 4.9 ± 1.8 mm, p<0.05) detected by a skinfold caliper.

The following Borg scale data were obtained after 20% and 60% MVC contractions respectively: females:11,6±1, males 11±0,5, p>0.05 and females 16,45±0,9, males 15,06±1,68. p>0.05.

A significant inter-gender difference was found for MVC (p<0.0001, Table 1) and for time of task at 60% MVC p<0,0001, Table 1).

**Table 1:** Elbow flexors maximal voluntary contraction (MVC, Kg) and time to task (TtT, s) in males and females. Data are expressed as mean±SD.

	MVC	TtT 60%
**Males**	45,84±8,6	38,82±10,1
**Females**	31,48±5,7***	53,3±17,4***

(Asterisk means “significantly different from males”:

*** = p < 0.0001)

Initial values of CV were higher in females than in males at both force levels (20% MCV: p<0.0001; 60% MCV: p<0.05, Table 2), whereas a lower initial estimate of FD was observed in females compared to males (20% MCV: p<0.05; 60% MCV: p<0.0001, Table 2).

**Table 2:** Means and standard deviations of CV, FD initial values (m/s) and slopes (%/s) at 20% MVC and 60% MVC and relative percentage of change (Δ%).

%MVC	CV	Males	Females	FD	Males	Females
**20%**	**Initial value**	4,18±0,45	4,79±0,89 ***	**Initial value**	1,59 ±0.02	1,58±0.02*
**60%**		4,69±0,50	5,41±1,68*		1,62±0.02	1,60±0.02**
**20%**	**Slope**	-0,03±0.04	-0.03±0.09	**Slope**	-0.02±0.02	-0.01±0.01
**60%**		-0.89±0.29	-0.66±0.41*		-0.22±0.07	-0.14±0.07*
**20%**	**Δ%**	-3,74±4,2	-2,75±8,8	**Δ%**	-1,69±2,1	-1,04±0,9
**60%**		-34,37±13	-32,15±14		-8,4±2,8	-7,2±3,4

(Asterisk means “significantly different from males”:

* = p<0,05;

** = p < 0.001;

*** = p <0.0001)

**Fig 2:**
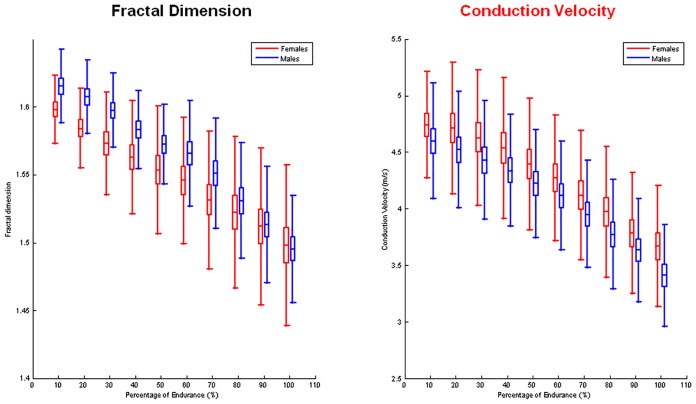
Graphs of the mean percentage of changes in FD (left) and CV (right) versus time in males and females during 60% MVC contraction. The time scale is expressed as a percentage of the total exhaustion time for each subject.

**Fig 3:**
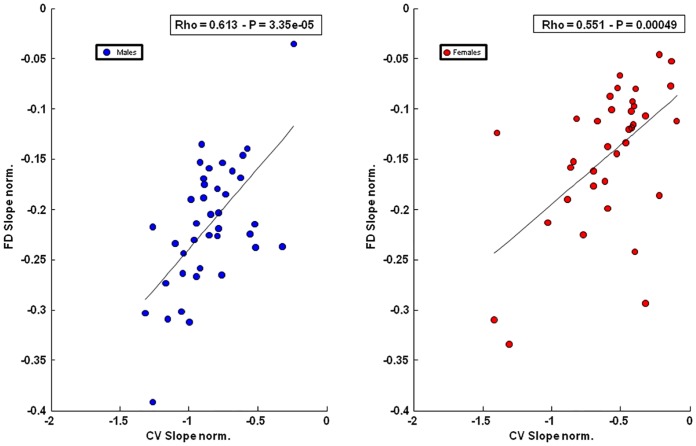
Scatter plots of the normalized slopes of FD versus CV for males (left) and females (right), at 60% of MVC isometric contraction.

No CV and FD slope difference was found at 20% MVC in both genders (CV: males -0.03%/s; females -0.03%/s; FD: male -0.02%/s; females -0.01%/s, p>0.05 Table 2). A significant slope difference was found at 60% MVC for both parameters and appeared significantly lower in females compared to males (CV: p<0.05; FD: p<0.0001, Table 2). No inter-gender difference was found in percentage change of CV and FD slopes at both levels of contraction (Table 2 and Fig 2). No statistical correlation was found between MVC and CV (males: r = 0,23; p = 0,14; females: r = 0,41; p = 0,011) or FD (males: r = 0,16; p = 0,3; females: r = 0,14; p = 0,39) initial estimates nor between MVC and CV (males: r = 0,01; p = 0,9; females: r = 0,23; p = 0,17) or FD (males: r = 0,01; p = 0,9; females: r = 0,3; p = 0,07) slopes at 60% MVC. No significant correlation was found between MVC and time of task at higher level of contraction for both genders (males: r = 0,17 p = 0,26; females: r = 0,26 p = 0,12; pooled data: r = 0,35, p = 0,001) and for time of task and slopes (FD males: r = 0,30, p = 0,05; females r = 0,25, p = 0,129; CV: males r = 0,03, p = 0,84; females r = 0,47, p = 0,003; pooled data: CV: r = 0,40, p = 0,0002; FD: r = 0,43, p = 0,0001). A significant positive correlation was found in both genders between CV and FD rate of changes measured during the sustained 60% MVC contraction (males: r = 0,613, p = 3.35e-05; females: r = 0,551, p = 0,00049) (Fig 3).

## Discussion

In the present study FD and CV measurements were used as myoelectric manifestations of muscle fatigue (central and peripheral) and were recorded by multichannel surface EMG during fatiguing isometric contractions of the biceps brachii. The most significant conclusion was that males showed a greater increase in the rate of change but no difference in the percentage change of CV and FD in comparison with females. Moreover, our results showed that gender and arm side do not have any significant interaction effect in the sEMG parameters measured.

These results show that gender slightly accounts for differences in myoelectric manifestations of peripheral fatigue, as mirrored by time changes in CV, and central fatigue, as mirrored by changes in FD/ MU synchronization.

### MVC

Males muscles are usually stronger than females [43]. This difference might be due to the presence of larger fibers, as observed both in the upper and lower limbs muscles, and not to differences in mode of fibers recruitment [44]. However, the mechanisms responsible for sex differences in fatigability are not dependent on MVC as no difference in time to failure has been observed in strength-matched males and females during sustained isometric submaximal contractions [37]. On account of this, we found that MVC was significantly higher in males than in females and no significant correlation was found between maximal torque and initial values and rate of change of measured myoelectric descriptors of fatigue.

### Initial values and of CV and FD

Based on Rainoldi’s observations, CV would be a good indicator of motor unit recruitment [45]. Initial values of CV are known to depend on muscle fibers recruitment, type of contraction [46], and fibers diameters [47]. Accordingly, our results showed that mean initial values of CV were higher during isometric contractions at 60% than at 20% MVC. This might be explained by the recruitment of progressively larger MU with increasing force output, and, therefore, with progressively higher CV values during fatiguing contractions, according to the Henneman size principle [48].

We observed with great interest that, at both force levels, initial values of CV were higher in females than in males with no interaction with arm side. These data, although in contrast with the presence of larger sized fibers in males than in females, agree with previous results obtained in our laboratory in vastus lateralis muscle [25] and may be explained considering that initial values of EMG variable estimates are not only related to the recruited MU pool, but are also affected by factors such as subcutaneous tissue thickness, skinfold thickness and fiber end effects [49,50]. Confirming to previously published data [51], we found that female biceps brachii was surmounted by a thicker skinfold than male one, probably causing overestimation of CV. In contrast, males reported higher FD initial values than females at both force levels analyzed. Although this difference may justify inter-gender differences in MU synchronization, caution should be used as it has been suggested that subject-dependent factors (e.g. fat layers and skin properties) may influence FD but not its rate of change [22]. Therefore initial values of FD not necessarily reflect the actual MU synchronization level whereas FD slope provides a reliable estimate of the actual change in MU synchronization.

### CV and FD slopes

**CV**: at 20% of MVC males and females presented an equal slope (around -0.03%/s, Table 2), consequently no statistically significant difference between genders was found, probably due to the low intensity level of contraction. At 60% MVC females displayed slower slope (-0.66±0,41%/s, Table 2) than males (-0.89±0,29%/s, Table 2) and the difference between genders appeared statistically significant (p<0,05). Differences in fibers size [52] and muscle composition [53,54], unlike subcutaneous thickness [50], may account for the observed inter-gender differences in peripheral sEMG descriptors of fatigue. Accordingly, it has been shown that a lower proportion of type II muscle fibers in females and a higher proportion of type I fibers in upper and lower limb muscles [44] may contribute to a lower decrease of CV over time compared to males [55,56,57]. Another aspect may concern blood flow because during low to moderate intensity sustained isometric contractions, when the muscle is not fully occluded, blood flow might be more restricted in males than in females at the same contraction intensity [58,59] due to their higher force generation [53,60].

**FD**: As previously reported, an increase in MU synchronization may be observed during fatiguing isometric contractions at 25% MVC or higher [11,25,26,61–65].

Accordingly, during 90s 20% MVC contraction no change in MU synchronization was observed in males and females, which showed similar FD rate of changes (-0.02%/s and -0.015/s, respectively, p>0.05, Table 2). We reckon that the level of contraction was insufficient to highlight gender differences due to task intensity. These conclusions are in concordance with the observation by Fallentin et al. [66] demonstrating that the patterns of motor unit recruitment during sub-maximal isometric contractions can be influenced by the level of contraction. The same authors highlighted the existence of a close relationship between intrinsic properties of the muscle and recruitment strategies of the central nervous system, which appear entirely different during static contractions at high or low intensities. Accordingly we found that at 60% MVC, FD slopes were lower in females than in males (-0.22%/s and -0.14%/s, respectively; p< 0.05, Table 2) probably suggesting a diverse level of MU synchronization at this contraction level.

### Time to task and percentage change in CV and FD

In agreement with previous investigations [31,60,67], we observed that at higher contraction percentage males had briefer endurance time than females. This difference may be attributed to the higher absolute target force in males compared to females.

In fact, a strong association between elbow flexors absolute force and time to task has been found and this relation appears to be exponential and independent from gender [67]. The shorter endurance time in stronger individuals has been attributed to their higher reflex-mediated adjustment of the mean arterial pressure during isometric contractions. This response, namely pressor response, is finalized to adjust the mismatch between muscular metabolic request and blood perfusion. Consistently a lower pressure response was found in females than males during submaximal contractions [67].

There was no sex difference in percentage change of CV and FD. Considering the difference in endurance time between males and females, our finding suggests that differences in the CV and FD rate of change probably occur as a result of differing times to task failure. These data reinforce the role of the absolute contraction intensity in determining the percentage change of myoelectric manifestations of fatigue at least at higher contraction levels [67]. However, electrophysiological adaptations of the skeletal muscle fibers leading to changes in myoelectric manifestations of fatigue may arise independently from changes in absolute torque have been reported following endurance training [68].

**Correlations**: No significant correlation was found between MVC or time to task and any initial values or slopes of CV and FD. Considering that the maximal voluntary strength that subjects can express may influence the time to task failure, these findings confirm the not so close relationship between time to task failure and sEMG descriptors of fatigue [67].

In agreement with previous findings from our laboratory [25,26], a significant positive correlation was found between FD and CV normalized slopes at 60% MVC in both genders. Considering that FD is less sensitive to changes in CV than other indices derived from the sEMG [22], we can assume that at higher level of contraction muscular fatigue may result of mutual interactions between central and peripheral mechanisms as previously hypothesized [69]. Moreover, a lower level of correlation found in females compared to males that may be explained by sex related differences in fatiguing threshold [70,71].

## Limitations of the study

The fatiguing isometric task used in the present study, although highly reliable for sEMG measurements, is far from real-life movement and everyday physical demands. A second limitation of the present study is that only one low intensity and one high intensity isometric sustained contraction was analyzed but, in our experimental conditions, a protocol setup consisting of contractions from 10 to 100% MVC, although much more exhaustive, would have been too long and unsustainable.

Finally Salomoni et al. recently evidenced the need to consider the effects of hormonal fluctuations in females when observing the effects of gender on muscle fatigue [72]. Therefore, in the present study females during menstrual cycle were excluded.

## Conclusions

This study investigated inter-gender differences in CV and, for the first time, FD slopes as indexes of peripheral and central myoelectric manifestations of fatigue during isometric contractions in healthy young humans. Results indicate that gender-related differences in fibers conduction velocity and MU synchronization rate of change may lie in disparities in strength. The correlation between FD and CV found at 60% MVC suggests that, during submaximal isometric contractions, central and peripheral myoelectric manifestation of fatigue mutually adapt to muscle fatigue in both genders.

## References

[1] Barry BK, Enoka RM (2007) The neurobiology of muscle fatigue: 15 years later. Integr Comp Biol 47: 465–473. 10.1093/icb/icm047 21672855

[2] Bigland-Ritchie B, Jones DA, Hosking GP, Edwards RH (1978) Central and peripheral fatigue in sustained maximum voluntary contractions of human quadriceps muscle. Clin Sci Mol Med 54: 609–614. 657729 10.1042/cs0540609

[3] Kent-Braun JA (1999) Central and peripheral contributions to muscle fatigue in humans during sustained maximal effort. Eur J Appl Physiol Occup Physiol 80: 57–63. 10.1007/s004210050558 10367724

[4] Schillings ML, Hoefsloot W, Stegeman DF, Zwarts MJ (2003) Relative contributions of central and peripheral factors to fatigue during a maximal sustained effort. Eur J Appl Physiol 90: 562–568. 10.1007/s00421-003-0913-4 12905050

[5] Merletti R, Bottin A, Cescon C, Farina D, Gazzoni M, Martina S, et al (2004) Multichannel surface EMG for the non-invasive assessment of the anal sphincter muscle. Digestion 69: 112–122. 10.1159/000077877 15087578

[6] De Luca CJ (1984) Myoelectrical manifestations of localized muscular fatigue in humans. Crit Rev Biomed Eng 11: 251–279. 6391814

[7] Enoka RM, Duchateau J (2008) Muscle fatigue: what, why and how it influences muscle function. J Physiol 586: 11–23. 10.1113/jphysiol.2007.139477 17702815 PMC2375565

[8] Bouissou P, Estrade PY, Goubel F, Guezennec CY, Serrurier B (1989) Surface EMG power spectrum and intramuscular pH in human vastus lateralis muscle during dynamic exercise. J Appl Physiol (1985) 67: 1245–1249. 2793717 10.1152/jappl.1989.67.3.1245

[9] Brody LR, Pollock MT, Roy SH, De Luca CJ, Celli B (1991) pH-induced effects on median frequency and conduction velocity of the myoelectric signal. J Appl Physiol (1985) 71: 1878–1885. 1761486 10.1152/jappl.1991.71.5.1878

[10] Komi PV, Tesch P (1979) EMG frequency spectrum, muscle structure, and fatigue during dynamic contractions in man. Eur J Appl Physiol Occup Physiol 42: 41–50. 499196 10.1007/BF00421103

[11] Holtermann A, Gronlund C, Karlsson JS, Roeleveld K (2009) Motor unit synchronization during fatigue: described with a novel sEMG method based on large motor unit samples. J Electromyogr Kinesiol 19: 232–241. 10.1016/j.jelekin.2007.08.008 18207421

[12] Semmler JG (2002) Motor unit synchronization and neuromuscular performance. Exerc Sport Sci Rev 30: 8–14. 11800501 10.1097/00003677-200201000-00003

[13] Datta AK, Farmer SF, Stephens JA (1991) Central nervous pathways underlying synchronization of human motor unit firing studied during voluntary contractions. J Physiol 432: 401–425. 1886061 10.1113/jphysiol.1991.sp018391 PMC1181332

[14] Bilodeau M, Arsenault AB, Gravel D, Bourbonnais D (1994) EMG power spectrum of elbow extensors: a reliability study. Electromyogr Clin Neurophysiol 34: 149–158. 8045246

[15] Dedering A, Roos af Hjelmsater M, Elfving B, Harms-Ringdahl K, Nemeth G (2000) Between-days reliability of subjective and objective assessments of back extensor muscle fatigue in subjects without lower-back pain. J Electromyogr Kinesiol 10: 151–158. 10818336 10.1016/s1050-6411(00)00009-2

[16] Gonzalez-Izal M, Malanda A, Gorostiaga E, Izquierdo M (2012) Electromyographic models to assess muscle fatigue. J Electromyogr Kinesiol 22: 501–512. 10.1016/j.jelekin.2012.02.019 22440555

[17] Kollmitzer J, Ebenbichler GR, Kopf A (1999) Reliability of surface electromyographic measurements. Clin Neurophysiol 110: 725–734. 10378745 10.1016/s1388-2457(98)00050-9

[18] Linssen WH, Stegeman DF, Joosten EM, van't Hof MA, Binkhorst RA, Notermans SL (1993) Variability and interrelationships of surface EMG parameters during local muscle fatigue. Muscle Nerve 16: 849–856. 10.1002/mus.880160808 8332138

[19] Merletti R, Fiorito A, Lo Conte LR, Cisari C (1998) Repeatability of electrically evoked EMG signals in the human vastus medialis muscle. Muscle Nerve 21: 184–193. 9466593 10.1002/(sici)1097-4598(199802)21:2<184::aid-mus5>3.0.co;2-7

[20] Ng JK, Richardson CA (1996) Reliability of electromyographic power spectral analysis of back muscle endurance in healthy subjects. Arch Phys Med Rehabil 77: 259–264. 8600868 10.1016/s0003-9993(96)90108-2

[21] Rainoldi A, Bullock-Saxton JE, Cavarretta F, Hogan N (2001) Repeatability of maximal voluntary force and of surface EMG variables during voluntary isometric contraction of quadriceps muscles in healthy subjects. J Electromyogr Kinesiol 11: 425–438. 11738955 10.1016/s1050-6411(01)00022-0

[22] Mesin L, Cescon C, Gazzoni M, Merletti R, Rainoldi A (2009) A bi-dimensional index for the selective assessment of myoelectric manifestations of peripheral and central muscle fatigue. J Electromyogr Kinesiol 19: 851–863. 10.1016/j.jelekin.2008.08.003 18824375

[23] Troiano A, Naddeo F, Sosso E, Camarota G, Merletti R, Mesin L (2008) Assessment of force and fatigue in isometric contractions of the upper trapezius muscle by surface EMG signal and perceived exertion scale. Gait Posture 28: 179–186. 10.1016/j.gaitpost.2008.04.002 18490165

[24] Kleine BU, Stegeman DF, Mund D, Anders C (2001) Influence of motoneuron firing synchronization on SEMG characteristics in dependence of electrode position. J Appl Physiol (1985) 91: 1588–1599. 11568140 10.1152/jappl.2001.91.4.1588

[25] Beretta-Piccoli M, D'Antona G, Barbero M, Fisher B, Dieli-Conwright CM, Clijsen R, et al (2015) Evaluation of central and peripheral fatigue in the quadriceps using fractal dimension and conduction velocity in young females. PLoS One 10: e0123921 10.1371/journal.pone.0123921 25880369 PMC4400165

[26] Boccia G, Dardanello D, Beretta-Piccoli M, Cescon C, Coratella G, Rinaldo N, et al (2016) Muscle fiber conduction velocity and fractal dimension of EMG during fatiguing contraction of young and elderly active men. Physiol Meas 37: 162–174. 10.1088/0967-3334/37/1/162 26684024

[27] Allen GM, Gandevia SC, McKenzie DK (1995) Reliability of measurements of muscle strength and voluntary activation using twitch interpolation. Muscle Nerve 18: 593–600. 10.1002/mus.880180605 7753121

[28] Bigland-Ritchie B, Johansson R, Lippold OC, Woods JJ (1983) Contractile speed and EMG changes during fatigue of sustained maximal voluntary contractions. J Neurophysiol 50: 313–324. 6308182 10.1152/jn.1983.50.1.313

[29] Gandevia SC, Allen GM, Butler JE, Taylor JL (1996) Supraspinal factors in human muscle fatigue: evidence for suboptimal output from the motor cortex. J Physiol 490 (Pt 2): 529–536. 8821149 10.1113/jphysiol.1996.sp021164 PMC1158689

[30] Kent-Braun JA, Le Blanc R (1996) Quantitation of central activation failure during maximal voluntary contractions in humans. Muscle Nerve 19: 861–869. 8965840 10.1002/(SICI)1097-4598(199607)19:7<861::AID-MUS8>3.0.CO;2-7

[31] Hunter SK, Critchlow A, Shin IS, Enoka RM (2004) Men are more fatigable than strength-matched women when performing intermittent submaximal contractions. J Appl Physiol (1985) 96: 2125–2132. 14966025 10.1152/japplphysiol.01342.2003

[32] Hunter SK (2014) Sex differences in human fatigability: mechanisms and insight to physiological responses. Acta Physiol (Oxf) 210: 768–789. 24433272 10.1111/apha.12234 PMC4111134

[33] Russ DW, Kent-Braun JA (2003) Sex differences in human skeletal muscle fatigue are eliminated under ischemic conditions. J Appl Physiol (1985) 94: 2414–2422. 12562681 10.1152/japplphysiol.01145.2002

[34] Martin PG, Rattey J (2007) Central fatigue explains sex differences in muscle fatigue and contralateral cross-over effects of maximal contractions. Pflugers Arch 454: 957–969. 10.1007/s00424-007-0243-1 17342531

[35] Farina D, Merletti R (2004) Methods for estimating muscle fibre conduction velocity from surface electromyographic signals. Med Biol Eng Comput 42: 432–445. 15320452 10.1007/BF02350984

[36] Keller ML, Pruse J, Yoon T, Schlinder-Delap B, Harkins A, Hunter SK (2011) Supraspinal fatigue is similar in men and women for a low-force fatiguing contraction. Med Sci Sports Exerc 43: 1873–1883. 21364478 10.1249/MSS.0b013e318216ebd4

[37] Yoon T, Schlinder Delap B, Griffith EE, Hunter SK (2007) Mechanisms of fatigue differ after low- and high-force fatiguing contractions in men and women. Muscle Nerve 36: 515–524. 10.1002/mus.20844 17626289

[38] Borg G (1982) Ratings of perceived exertion and heart rates during short-term cycle exercise and their use in a new cycling strength test. Int J Sports Med 3: 153–158. 10.1055/s-2008-1026080 7129724

[39] Merletti R, Roy S (1996) Myoelectric and mechanical manifestations of muscle fatigue in voluntary contractions. J Orthop Sports Phys Ther 24: 342–353. 10.2519/jospt.1996.24.6.342 8938600

[40] Gitter JA, Czerniecki MJ (1995) Fractal analysis of the electromyographic interference pattern. J Neurosci Methods 58: 103–108. 7475215 10.1016/0165-0270(94)00164-c

[41] Farina D, Merletti R (2003) A novel approach for estimating muscle fiber conduction velocity by spatial and temporal filtering of surface EMG signals. IEEE Trans Biomed Eng 50: 1340–1351. 10.1109/TBME.2003.819847 14656063

[42] Andreassen S (1987) Methods for computer-aided measurement of motor unit parameters. Electroencephalogr Clin Neurophysiol Suppl 39: 13–20. 3477421

[43] Hunter SK (2013) Sex differences in human fatigability: mechanisms and insight to physiological responses. Acta Physiol (Oxf) 210: 768–789. 10.1111/apha.12234 PMC4111134 24433272

[44] Miller AE, MacDougall JD, Tarnopolsky MA, Sale DG (1993) Gender differences in strength and muscle fiber characteristics. Eur J Appl Physiol Occup Physiol 66: 254–262. 8477683 10.1007/BF00235103

[45] Rainoldi A, Galardi G, Maderna L, Comi G, Lo Conte L, Merletti R (1999) Repeatability of surface EMG variables during voluntary isometric contractions of the biceps brachii muscle. J Electromyogr Kinesiol 9: 105–119. 10098711 10.1016/s1050-6411(98)00042-x

[46] Bernardi M, Solomonow M, Baratta RV (1997) Motor unit recruitment strategy of antagonist muscle pair during linearly increasing contraction. Electromyogr Clin Neurophysiol 37: 3–12. 9063656

[47] Blijham PJ, ter Laak HJ, Schelhaas HJ, van Engelen BG, Stegeman DF, Zwarts MJ (2006) Relation between muscle fiber conduction velocity and fiber size in neuromuscular disorders. J Appl Physiol (1985) 100: 1837–1841. 16424073 10.1152/japplphysiol.01009.2005

[48] Adam A, De Luca CJ (2003) Recruitment order of motor units in human vastus lateralis muscle is maintained during fatiguing contractions. J Neurophysiol 90: 2919–2927. 10.1152/jn.00179.2003 14615422

[49] Farina D, Macaluso A, Ferguson RA, De Vito G (2004) Effect of power, pedal rate, and force on average muscle fiber conduction velocity during cycling. J Appl Physiol (1985) 97: 2035–2041. 15286050 10.1152/japplphysiol.00606.2004

[50] Merletti R, Farina D (2016) Biophysics of the Generation of EMG Signals Surface Electromyography: Physiology, Engineering and Applications: Wiley-IEEE press pp. 30–53.

[51] Inglis JG, Vandenboom R, Gabriel DA (2013) Sex-related differences in maximal rate of isometric torque development. J Electromyogr Kinesiol 23: 1289–1294. 10.1016/j.jelekin.2013.09.005 24148962

[52] Simoneau JA, Bouchard C (1989) Human variation in skeletal muscle fiber-type proportion and enzyme activities. Am J Physiol 257: E567–572. 2529775 10.1152/ajpendo.1989.257.4.E567

[53] Hunter SK (2009) Sex differences and mechanisms of task-specific muscle fatigue. Exerc Sport Sci Rev 37: 113–122. 10.1097/JES.0b013e3181aa63e2 19550202 PMC2909485

[54] Roepstorff C, Thiele M, Hillig T, Pilegaard H, Richter EA, Wojtaszewski JF, et al (2006) Higher skeletal muscle alpha2AMPK activation and lower energy charge and fat oxidation in men than in women during submaximal exercise. J Physiol 574: 125–138. 10.1113/jphysiol.2006.108720 16600998 PMC1817798

[55] Sadoyama T, Masuda T, Miyata H, Katsuta S (1988) Fibre conduction velocity and fibre composition in human vastus lateralis. Eur J Appl Physiol Occup Physiol 57: 767–771. 3416864 10.1007/BF01076001

[56] Kupa EJ, Roy SH, Kandarian SC, De Luca CJ (1995) Effects of muscle fiber type and size on EMG median frequency and conduction velocity. J Appl Physiol (1985) 79: 23–32. 7559225 10.1152/jappl.1995.79.1.23

[57] Mannion AF, Dumas GA, Stevenson JM, Cooper RG (1998) The influence of muscle fiber size and type distribution on electromyographic measures of back muscle fatigability. Spine (Phila Pa 1976) 23: 576–584. 9530789 10.1097/00007632-199803010-00010

[58] Barnes WS (1980) The relationship between maximum isometric strength and intramuscular circulatory occlusion. Ergonomics 23: 351–357. 10.1080/00140138008924748 7202390

[59] Sadamoto T, Bonde-Petersen F, Suzuki Y (1983) Skeletal muscle tension, flow, pressure, and EMG during sustained isometric contractions in humans. Eur J Appl Physiol Occup Physiol 51: 395–408. 6685038 10.1007/BF00429076

[60] Hunter SK, Schletty JM, Schlachter KM, Griffith EE, Polichnowski AJ, NG AV (2006) Active hyperemia and vascular conductance differ between men and women for an isometric fatiguing contraction. J Appl Physiol (1985) 101: 140–150. 16601303 10.1152/japplphysiol.01567.2005

[61] Arjunan SP, Kumar DK, Naik G (2014) Computation and evaluation of features of surface electromyogram to identify the force of muscle contraction and muscle fatigue. Biomed Res Int 2014: 197960 10.1155/2014/197960 24995275 PMC4065755

[62] Contessa P, Adam A, De Luca CJ (2009) Motor unit control and force fluctuation during fatigue. J Appl Physiol (1985) 107: 235–243. 19390005 10.1152/japplphysiol.00035.2009 PMC2711782

[63] Kumar DK, Arjunan SP, Naik GR (2011) Measuring increase in synchronization to identify muscle endurance limit. IEEE Trans Neural Syst Rehabil Eng 19: 578–587. 10.1109/TNSRE.2011.2163527 21846609

[64] Semmler JG, Steege JW, Kornatz KW, Enoka RM (2000) Motor-unit synchronization is not responsible for larger motor-unit forces in old adults. J Neurophysiol 84: 358–366. 10899210 10.1152/jn.2000.84.1.358

[65] Talebinejad M, Chan AD, Miri A (2010) Fatigue estimation using a novel multi-fractal detrended fluctuation analysis-based approach. J Electromyogr Kinesiol 20: 433–439. 10.1016/j.jelekin.2009.06.002 19589697

[66] Fallentin N, Jorgensen K, Simonsen EB (1993) Motor unit recruitment during prolonged isometric contractions. Eur J Appl Physiol Occup Physiol 67: 335–341. 8299601 10.1007/BF00357632

[67] Hunter SK, Enoka RM (2001) Sex differences in the fatigability of arm muscles depends on absolute force during isometric contractions. J Appl Physiol (1985) 91: 2686–2694. 11717235 10.1152/jappl.2001.91.6.2686

[68] Vila-Cha C, Falla D, Farina D (2010) Motor unit behavior during submaximal contractions following six weeks of either endurance or strength training. J Appl Physiol (1985) 109: 1455–1466. 20829500 10.1152/japplphysiol.01213.2009

[69] Nybo L, Secher NH (2004) Cerebral perturbations provoked by prolonged exercise. Prog Neurobiol 72: 223–261. 10.1016/j.pneurobio.2004.03.005 15142684

[70] Clark BC, Manini TM, The DJ, Doldo NA, Ploutz-Snyder LL (2003) Gender differences in skeletal muscle fatigability are related to contraction type and EMG spectral compression. J Appl Physiol (1985) 94: 2263–2272. 12576411 10.1152/japplphysiol.00926.2002

[71] Bazzucchi I, Felici F, Macaluso A, De Vito G (2004) Differences between young and older women in maximal force, force fluctuations, and surface EMG during isometric knee extension and elbow flexion. Muscle Nerve 30: 626–635. 10.1002/mus.20151 15389720

[72] Salomoni S, Soares FA, de Oliveira Nascimento FA, da Rocha AF (2008) Gender differences in muscle fatigue of the biceps brachii and influences of female menstrual cycle in electromyography variables. Conf Proc IEEE Eng Med Biol Soc 2008: 2598–2601. 10.1109/IEMBS.2008.4649732 19163235

